# Comparative Analysis of Intravenous Immunoglobulins (IVIg) vs Plasmapheresis (PLEX) in the Management of Myasthenic Crisis

**DOI:** 10.7759/cureus.68895

**Published:** 2024-09-07

**Authors:** Ahmad Zain, Muhammad Shariq Akram, Fatima Ashfaq, Armghan Ans, Hasaan H Ans

**Affiliations:** 1 Internal Medicine, Services Hospital Lahore, Lahore, PAK; 2 Internal Medicine, King Edward Medical University (KEMU), Lahore, PAK; 3 Radiology, Stanford University, Palo Alto, USA; 4 Internal Medicine, Services Institute of Medical Sciences, Lahore, PAK; 5 Neurology, Medical College of Wisconsin, Milwaukee, USA; 6 Vascular Neurology, University of Pittsburgh Medical Center, Pittsburgh, USA; 7 Internal Medicine, FMH College of Medicine & Dentistry, Lahore, PAK

**Keywords:** intravenous immunoglobulin (ivig), myasthenia gravis crisis, myasthenia gravis exacerbation, myasthenia gravis (mg), plasmapharesis

## Abstract

Myasthenia gravis (MG) is an autoimmune disorder affecting postsynaptic membranes in neuromuscular junctions, presenting as fatigable muscle weakness. Myasthenic crisis is a life-threatening complication characterized by severe respiratory insufficiency necessitating invasive or noninvasive ventilation. Two rapid therapies used to manage myasthenic crises are intravenous immunoglobulins (IVIg) and plasmapheresis (PLEX). Their comparative effectiveness remains equivocal. Our article examines evidence from several clinical trials and observational studies, in order to determine the superiority of one treatment over the other. Multiple factors can complicate the choices between two treatments. We concluded that the choice between PLEX and IVIg is multifaceted, guided by individual patient characteristics, institutional resources, and clinician preference. While PLEX can be considered as first-line for rapid clinical outcomes, it is hard to pick one treatment over the other, and careful consideration of comorbidities and resource availability is crucial. Our article highlights the need for further research to establish definitive guidelines and enhance patient outcomes in myasthenic crisis patients.

## Introduction and background

Myasthenia gravis is a progressive autoimmune disorder characterized by fatigable weakness of ocular, bulbar, respiratory, and limb muscles, caused by autoantibodies, primarily against postsynaptic nicotinic acetylcholine receptors (AchR), leading to a disruption in neuromuscular transmission [1, 2]. It is the most common disorder of neuromuscular junction. The incidence of acquired myasthenia gravis ranges from 0.3-2.8 percent per 100,000 and a total of 700,000 people are estimated to be affected worldwide [1]. The incidence follows a bimodal distribution with a female predominance in the early-onset group (before 50) and a male predominance in the late-onset group (after 50) [1,2]. The most common antibodies detected in myasthenia gravis are against presynaptic nicotinic acetylcholine receptors (AchR), however, antibodies against muscle-specific tyrosine kinase (anti MuSK antibodies), or Lipoprotein receptor-related protein 4 (Lrp-4) can also be detected [2]. Seronegative myasthenia gravis refers to the cases of myasthenia in which no antibodies are detectable in the serum. The treatment options for myasthenia gravis have evolved significantly over time with advancements in immune modulation, and now several treatment options are available at every stage of the disease [3].

## Review

Myasthenic crisis

Myasthenic crisis is defined as worsening respiratory and bulbar muscle weakness from myasthenia gravis that requires intubation or non-invasive ventilation [1]. It is life-threatening and can be triggered by factors such as fever, surgery, pregnancy, emotional stress, and systemic illnesses [4,5]. Impending myasthenic crisis as the name implies is worsening of myasthenia gravis that will progress to myasthenic crisis in days to weeks. Diagnosis of myasthenic crisis is mainly clinical and suggestive signs/symptoms include dyspnea, dysphagia, and signs of respiratory distress such as shallow breathing, poor respiratory effort inability, to speak in full sentences, and use of accessory respiratory muscles [6,7]. Measures of respiratory muscle function such as forced vital capacity (FVC), and negative inspiratory force (NIF) at bedside, can help monitor the situation and guide the next steps in the management [4]. Myasthenic crisis is a true neurologic emergency and prompt identification and early management of symptoms is crucial in such patients. It is important to differentiate between myasthenic crisis and ‘cholinergic crisis’ because both can resemble clinically. In general concept, myasthenic crisis is caused by under-stimulation of neuromuscular junction caused by worsening myasthenia gravis, whereas cholinergic crisis is caused by over-stimulation of neuromuscular junction with use of acetylcholinesterase inhibitors for symptomatic relief, such as pyridostigmine. In addition to bulbar and respiratory insufficiency, symptoms of cholinergic excess are seen with cholinergic crisis, such as abdominal cramps, nausea, diarrhea, urinary frequency, salivation, lacrimation, bradycardia, and hypotension [3,4].

Management of myasthenic crisis

Management of a myasthenic crisis involves intensive care unit admission, assessing the need for intubation or non-invasive ventilation, and initiation of rapid therapy with intravenous immunoglobulins (IVIg) or plasmapheresis (PLEX). We will focus on rapid therapies with IVIg or PLEX in the remaining part of the article. 

Currently, plasma exchange (PLEX) and intravenous immunoglobulins (IVIg) are utilized as rapid therapies [5, 6]. Despite agreement among physicians on the use of these treatments, there is no universally accepted standard of care due to the heterogeneity of this condition and the difference in availability of resources, which presents a limitation in determining a single optimal treatment for all patients [1].

Intravenous immunoglobulins (IVIg)

Intravenous immunoglobulins (IVIg) are derived from human plasma and administered intravenously. The exact mechanism of action of IVIg is complex and partially understood, but overall IVIg works by downregulation of pathogenic immune responses. Several mechanisms involve the modulation of autoantibodies, inhibition of the complement system, disruption of membrane attack complex (MAC) formation, and modulation of cytokines and cell-mediated immunity [4,6]. In recent years, there has been a notable rise in the utilization of intravenous immunoglobulins (IVIg) for the management of MG exacerbation. Several factors contribute to this upward trend, including the convenience of administration compared to plasma exchange (PLEX) and the favorable long-term effects observed in individuals without significant comorbid medical conditions such as chronic kidney disease (CKD), diabetes, hypertension, and hypercoagulable states [4]. The standard dosage of IVIg involves administering 0.4g/kg body weight over a span of 3-5 days [6]. IVIg therapy is generally associated with mild adverse effects, such as headaches, chills, myalgia, and temporary hypertension. These effects are primarily linked to the early or rapid initiation of therapy [8, 9].

Plasmapheresis (PLEX)

Plasmapheresis is a technique that involves extracorporeal removal, return, or exchange of blood plasma using the technique of either centrifugation (based on differential gravities of molecules) or membrane filtration (based on differential sizes of molecules) [10,11]. One of the key differences between these two techniques is that in centrifugation, the filtered plasma is wasted, and donor plasma and colloids are used as a replacement, while in membrane filtration, the filtered plasma is returned to the patient as undesirable molecules can be selectively filtered across the membrane. Typically, the standard procedure involves 5-6 sessions on alternate days, with a daily volume of 2-4 liters [12, 13]. A more recent technique involves antigen-specific immunoadsorption. Although clinical studies have demonstrated comparable outcomes among different techniques, immune adsorption offers the advantage of improved tolerability by avoiding the need for the replacement of lost components [10,14]. Plasmapheresis requires central venous access which can be a limiting factor among others to utilize the procedure. 

Plasmapheresis (PLEX) vs intravenous immunoglobulins (IVIg):

In clinical research trials, disease-specific measures are used to assess the response to therapies in patients including IVIg and PLEX. These measures encompass functional improvement reported by patients and quality of life measures [15]. Examples of these disease-specific measures include the Quantitative MG score (MGFA-QMG), MG-Activities of Daily Living profile (MG-ADL), manual muscle test (MG-MMT), MG-Composite (MG-C), and Quality of Life 15 (MG-QoL15). However, data on the use of these measures for evaluating the outcomes of PLEX vs IVIg therapies is limited.

In the past, specialists have generally viewed Intravenous immune globulin (IVIg) and plasma exchange (PLEX) as equally effective treatments for severe myasthenia gravis [16, 17, 18]. Nevertheless, the selection between PLEX and IVIG hinges on a variety of factors, encompassing individual patient characteristics, institutional experience, accessibility, and established practices [19].

Gajdos et al. [17] conducted a randomized controlled trial in 1997 to determine the efficacy and tolerability of PLEX or IVIg in myasthenia gravis exacerbation patients. The trial used variation in Myasthenic Muscular Score (MSS) at day 15 as the primary endpoint. A sample of 87 patients was selected, 41 patients were randomized to receive three sessions of PLEX, and 46 patients were randomized to receive 0.4 g/kg of IVIg further subdivided into two groups receiving 3 days (n=23) or 5 days (n=23) course of IVIg. The trial revealed no significant difference in efficacy between the two groups (IVIg vs PLEX). There were 14 total side effects observed in nine patients, eight in the PLEX group and one in the IVIg group, thus showing better tolerability in the IVIg group. Out of eight patients with side effects observed in the PLEX group, two side effects were hemodynamically significant and included retroperitoenal hematoma and femoral vein thrombosis (in separate patients) and required discontinuation of PLEX, while in the IVIg group, headache was observed in one patient. The study showed that the 5-day IVIg schedule was not superior to a 3-day schedule. The study was the first randomized clinical trial comparing the efficacy of both treatments; however, it had its limitations of a smaller sample size and an inability to assess the long term effects of both treatments as the primary endpoint and follow up was limited to 15 days.

Subsequently, in 1999, Qureshi et al. [20] conducted a retrospective multicentric study comparing the efficacy and tolerance of PLEX and IVIg. The study analyzed the treatment of 54 episodes of myasthenic crisis and looked at the ventilatory status of patients at 2 weeks (including ability to extubate), and functional outcome at 1 month period. Myasthenic crisis was described by acute respiratory muscle weakness (defined as forced vital capacity FVC ≤ 1.0 liter, negative inspiratory force NIF ≤ 20 cm H2O, or a need for mechanical ventilation. The choice of treatment was based on physician preferences and 26 episodes (24 patients) were treated with IVIg and 28 episodes (27 patients) received PLEX. The patients in the PLEX group had 5-6 sessions performed on alternate days. The patients in the IVIG group received 400 mg/kg/day of IVIg for a total of 5 days. The study demonstrated that plasmapheresis yielded superior outcomes compared to IVIg in terms of extubation at 2 weeks and functional outcomes at 1 month. However, the complication rate was higher with PLEX compared to IVIg and complications observed were of cardiovascular and infectious nature.

Most recently, in 2022, Wang et al. [21] conducted a prospective cohort study involving 40 myasthenic crisis patients who were Acetylcholine receptor antibody (AchRAb) positive. 12 patients received treatment with PLEX vs 28 patients receiving IVIg. The primary outcome was a change in MGFA-QMG score at 1 week from the crisis at 1 month and at 2 months off ventilation. Their findings indicated that plasmapheresis was associated with a shorter ICU stay and a more rapid clinical improvement. However, clinical efficacy after one month was found to be similar with both treatments. IVIg treatment was associated with a significant decrease in acetylcholine receptor (AchR) antibody titer.

Of note, Stricker et al. [22] in 1993 documented four patients who did not respond to IVIG but subsequently improved on PLEX. PLEX sessions were administered within 48 hours of the last IVIg treatment. This study potentially highlights the rapid improvement of symptoms with PLEX as compared to IVIg and may not establish the superiority of one treatment vs the other in terms of overall efficacy, even though it originally postulated that PLEX might be superior to IVIg for the treatment of myasthenic crisis in certain patients.

D. Barth et al. in 2010 [16] conducted a randomized, evaluator-masked study in patients with moderate to severe myasthenia gravis comparing the efficacy of IVIg vs PLEX. The study consisted of 84 patients with moderate to severe myasthenia gravis defined by a Quantitative Myasthenia Gravis Score (QMGS) of >10.5 and a worsening weakness. The diagnosis of myasthenia gravis was based on clinical judgment, single-fiber electromyography (EMG) results, and abnormal repetitive nerve stimulation tests. Patients were not excluded if they were seronegative and the presence of AchRAb and anti-MuSK antibodies were taken as a supportive finding for the diagnosis. Worsening weakness was defined by worsening ptosis, blurry vision, diplopia, dysarthria, dysphagia, worsening respiratory status, fatiguability, and limb weakness. Patients with active renal or hepatic disease, cardiac disease, hypercoagulable state, history of anaphylaxis to IVIg, uncontrolled hypertension, pregnancy, or breastfeeding were excluded from the study. Patients were randomized to treatment groups including either IVIg or PLEX. IVIg group received 1g/kg/day IVIg for consecutive 2 days. PLEX group received five plasma exchange sessions in total performed every second day. The primary outcome was a change in QMGS score at 14 days after full treatment and secondary outcomes included changes in rapid nerve stimulation tests, single fiber EMG results, AchRAb titers, need for positive pressure ventilation or intubation, and need for ICU admission. The results of this study provided class 1 evidence of comparable efficacy with 1 g/kg/day IVIg or five sessions of PLEX as both IVIg and PLEX reduced the QMGS scores for moderate to severe myasthenia gravis patients. The presence of baseline worse clinical status and positive AchRAb titers suggested a better response to both therapies. It is worth noting that the study included patients with moderate to severe myasthenia gravis patients and not just myasthenic crisis patients.

Murthy et. al [23] conducted a retrospective study of 23 episodes of myasthenic crisis that were either treated with PLEX or IVIg. Myasthenic crisis was defined as a weakness from acquired myasthenia gravis that required intubation. Infections were identified as the most common precipitating factor for a myasthenic crisis. Intubation criteria included vital capacity <15 ml/kg, widening of A-a gradient, inability to clear secretions, weak cough, and nasal voice. The primary outcome was the number of days required for disease stabilization which was measured by the time taken for extubation of the patient. Extubation criteria included partial pressure of oxygen (PaO2)> 80 mmHg on a fraction of inspired oxygen (FiO2) of 40%, partial pressure of carbon dioxide (PCO2)<42 mmHg, and a spontaneous breathing rate <20. The choice of PLEX or IVIg was made based on accessibility and affordability. 15 episodes were treated with small volume plasma exchange and the PLEX group received a total of five sessions performed on alternate days, each session consisted of two sittings, and a maximum of 500 ml of plasma exchange was performed per sitting. Eight episodes were treated with IVIg, and the IVIg group received 400 mg/kg/day of IVIg administered for 5 days. The time taken for disease stabilization, the median number of days to extubate was 8 days in the PLEX group and 10 days in the IVIg group. One person in the PLEX group developed hypotension, one person in the IVIG group developed temporary elevation of BUN and creatinine, while one person in the IVIG group could not be extubated and died. In conclusion, this retrospective study showed that both IVIg and PLEX have equal efficacy in terms of disease stabilization measured by the number of days to extubate.

Pavlekovics et al. [24] in 2023 conducted a systematic review and meta-analysis of the efficacy and safety of PLEX vs IVIg in moderate to severe myasthenia gravis with a special focus on a faster relapse control, which to our knowledge is the first meta-analysis of RCT studies. This systematic review had strict criteria and three RCTs met the inclusion criteria and two RCTs investigating a total of 114 patients qualified for meta-analysis to study safety and efficacy. Moderate myasthenia gravis was defined as a QMGS score of 10-16 points while severe myasthenia gravis was defined as a QMG score of more than 16 points. The study showed that there was no significant difference between the two treatments in terms of side effect profiles, and PLEX may have a quicker beneficial effect at 2 weeks, as shown by the difference of QMG score of more than 2.5 points improvement with PLEX as compared to IVIg at 2 weeks mark.

There are certain limitations associated with plasmapheresis, such as the extensive venous access required and the inherent risks of hemodynamic instability. Furthermore, the comparatively high cost, invasive nature, and potential adverse reactions to anticoagulants, including citrate, serve as additional factors restricting the utilization of PLEX [25, 26, 27]. 

IVIg treatment has some caveats as well like the regional accessibility, and cost of treatment, however, it does not require extensive venous access and administration is relatively simpler. The selection between PLEX and IVIg can be influenced by individual cases, for instance, PLEX is contraindicated in cases of sepsis, while IVIg is contraindicated in IgA deficiency and is not recommended for chronic kidney disease (CKD) patients [4].

There is no sufficient data and research to establish definitive guidelines for the utilization of PLEX vs. IVIg [17, 18, 20]. The choice of rapid treatment of myasthenic crisis with PLEX or IVIg ultimately is based on the individual clinical picture, availability of resources, anecdotal evidence, and institutional preferences.

Cost comparison of IVIg vs PLEX

When selecting the treatment modality for the acute myasthenic crisis, cost should never be the main factor, however, these cost comparison studies highlight the areas in which cost can be reduced for the sake of overall optimization of the healthcare system. Heatwole et al. [28] in 2011 conducted an itemized comparative cost-minimization analysis of IVIg vs PLEX. Several factors were considered to calculate the costs including the initial cost of therapy, length of therapy, location and cost of hospitalization of each therapy, and cost of treatment of complications associated with the therapy. The results of this study concluded that IVIG may be a short-term cost-minimizing therapy compared to PLEX with an average savings of $22,326. Overall costs were heavily dependent on IVIg dosing, length of hospital stay, and number of PLEX sessions. On the other hand, Furlan et al. [29] conducted a cost-minimization analysis comparing IVIG and PLEX for the treatment of myasthenia exacerbations. The study combined Ontario-based health cost data with clinical data from an RCT by Barth D et al. [16] and analyzed the cost from the perspective of a public healthcare insurer and from the perspective of a tertiary university hospital. The study concluded that PLEX can be a short-term cost minimalizing therapy as compared to IVIG in patients with BMI<15.7 kg/m2 from the perspective of a public healthcare insurer.

## Conclusions

Myasthenia gravis is an autoimmune disorder of the neuromuscular junction characterized by antibodies primarily against the acetylcholine receptor (AchR) on the postsynaptic membrane leading to fatigable muscle weakness. Myasthenic crisis is a life-threatening medical emergency in a myasthenia gravis patient characterized by respiratory insufficiency that may require invasive or noninvasive ventilation. Apart from ventilatory support, rapid therapies like plasmapheresis (PLEX) and intravenous immunoglobulins (IVIg) are required to treat the symptoms. Both PLEX and IVIg have their pros and cons. In general, PLEX is associated with a rapid improvement of clinical outcomes however the efficacy of both treatment options over the long term is comparable, and there is no sufficient data to establish the superiority of one option over the other. The decision to choose IVIg or PLEX should be made based on individual cases and the best use of resources.
